# Duplex Doppler Measurement of Renal Resistive Index in Non-Dialysis Chronic Kidney Disease Outpatients: A Proposed Standardized Protocol

**DOI:** 10.3390/diagnostics16152438

**Published:** 2026-08-01

**Authors:** GianLuca Colussi, Lisa Giusto, Alessandra Rigamonti, Stefania Rondinella, Giulia Marta Viglione, Marco Fabio Cola, Piergiorgio Gaudenzi, Maurizio Tonizzo, Giulio Romano

**Affiliations:** 1 Internal Medicine, Department of Medical Sciences, University of Ferrara, 44121 Ferrara, Italy; 2 Hypertension Unit, Division of Internal Medicine, Department of Medicine, Azienda Ospedaliero-Universitaria “Sant’Anna”, 44124 Ferrara, Italy; 3 Vascular Ultrasound Unit, Division of Internal Medicine, Department of Medicine, Azienda Ospedaliero-Universitaria “Sant’Anna”, 44124 Ferrara, Italy; 4 Department of Radiology, Azienda Sanitaria Universitaria Friuli Centrale (ASUFC) “Santa Maria della Misericorida”, 33100 Udine, Italy; 5 Vascular Ultrasound and Hypertension Unit, Division of Internal Medicine, Department of Medicine, Azienda Sanitaria Friuli Occidentale (ASFO), 33170 Pordenone, Italy; 6 Nephrology, Department of Medicine, University of Udine, 33100 Udine, Italy; giulio.romano@uniud.it

**Keywords:** kidney ultrasound, nephrovascular disease, cardiovascular disease, renal failure, hemodynamics, renal resistive index

## Abstract

The renal resistive index (RRI) is a Doppler-derived parameter influenced by intrarenal hemodynamics, vascular compliance, pulsatile load, and systemic cardiovascular factors. In outpatient nephrology practice, RRI may provide adjunctive hemodynamic and prognostic information when interpreted together with kidney morphology, estimated glomerular filtration rate, proteinuria, blood pressure, heart rate, rhythm, and cardiovascular context. This protocol aims to standardize duplex Doppler acquisition, calculation, interpretation, and reporting of RRI in outpatient adults with native-kidney chronic kidney disease (CKD). The protocol defines patient eligibility and preparation, same-visit clinical data collection, grayscale kidney assessment, color Doppler localization of intrarenal arteries, pulsed-wave Doppler settings, bilateral upper-, middle-, and lower-pole sampling, waveform validity criteria, RRI calculation rules, image archiving, and structured reporting. Technical pitfalls and hemodynamic confounders are incorporated into the workflow to support reproducibility and serial comparability. The expected output is a standardized RRI report including kidney-specific and bilateral mean RRI values when minimum validity criteria are met, the number and location of valid sampling sites, grayscale morphologic findings, same-visit hemodynamic context, relevant technical limitations, and interpretive caveats. The protocol is designed to reduce acquisition variability and improve the consistency of RRI documentation in outpatient CKD practice. RRI should not be interpreted as a stand-alone diagnostic test or direct surrogate of renal vascular resistance. A standardized acquisition and reporting protocol may improve reproducibility and allow RRI to be used more appropriately as an adjunctive hemodynamic and prognostic marker in adults with non-dialysis-dependent CKD.

## 1. Introduction

The renal resistive index (RRI) is calculated from spectral Doppler measurements of peak systolic velocity (PSV) and end-diastolic velocity (EDV), applying the Pourcelot’s formula (PSV − EDV)/PSV [1,2]. Although RRI was thought to be a direct measure of intrarenal vascular resistance [3], additional research has shown that RRI serves as an integrated measure of arterial compliance and pulsatile load [4,5]. As a result, RRI represents a complex interaction between renal microcirculation and systemic hemodynamics, which is significantly affected by factors such as pulse pressure, vascular compliance, arterial stiffness, age, and cardiac function [6,7,8,9].

RRI may change over short time scales in response to renal or systemic hemodynamic variation, but its behavior depends on the relative contribution of vascular compliance, pulsatile load, preglomerular tone, and structural vascular remodeling [4,5,6]. Intervention studies in healthy subjects and selected clinical settings, including primary aldosteronism, support the view that reversible vasomotor tone may influence RRI [6,10]. Conversely, in conditions characterized by structural microvascular remodeling, such as systemic sclerosis, RRI may be less responsive to short-term vasoactive intervention, suggesting that fixed vascular and parenchymal changes can dominate over reversible vasomotor effects [11]. For these reasons, RRI should be interpreted together with patients’ clinical history, kidney morphology, proteinuria, estimated glomerular filtration rate (GFR), and same-visit hemodynamic conditions.

Chronic kidney disease (CKD) is defined according to Kidney Disease: Improving Global Outcomes (KDIGO) criteria as persistent abnormalities of kidney structure or function for more than 90 days, with implications for health [12]. In CKD, RRI has been associated in some studies with disease severity, histologic nephroparenchymal damage and chronicity, cardiovascular burden, and adverse renal outcomes [5,13,14]. However, RRI lacks etiologic specificity and should not be used to define CKD cause or stage. Therefore, the RRI’s most plausible role in outpatient practice is as an adjunctive hemodynamic marker that may enrich clinical interpretation when integrated with structural kidney assessment and standard CKD risk markers [5,13,15]. In addition, RRI prognostic value has become increasingly well established, since various cohorts of CKD patients have reported associations between elevated RRI and the progression of kidney dysfunction or all-cause mortality [16,17,18,19].

Although the physiological determinants and clinical associations of RRI have been described in vascular ultrasonography literature, nephrology sonography curricula, and technical Doppler reviews [5,20,21,22,23,24], the practical elements required for reproducible outpatient measurement are rarely synthesized in a single publication. In particular, there is a need for an operational protocol that integrates patient preparation, same-visit hemodynamic documentation, grayscale kidney assessment, intrarenal vessel selection, Doppler optimization, bilateral multi-site sampling, waveform validity criteria, calculation rules, troubleshooting, and structured reporting. The added value of the present protocol is therefore not the introduction of a new Doppler index, but the consolidation of dispersed technical and interpretive information into a reproducible workflow for adults with native-kidney non-dialysis-dependent CKD. This protocol aims to standardize RRI acquisition, documentation, and interpretation in outpatient CKD practice while preserving the appropriate role of RRI as an adjunctive hemodynamic and prognostic marker rather than a stand-alone diagnostic test [5,13,15,16,17,18,19].

## 2. Experimental Design

### 2.1. Protocol Development and Scope of Standardization

This protocol was developed as a pragmatic technical workflow through a targeted synthesis of renal Doppler/RRI literature, nephrology sonography curricula, Doppler methodology reviews, previously published outpatient CKD RRI cohorts, and the authors’ multidisciplinary experience in renal and vascular ultrasound. Its recommendations draw on three complementary sources: direct renal Doppler/RRI evidence for RRI calculation, intrarenal arterial sampling, spectral Doppler optimization, waveform validity, reproducibility, and interpretation [3,4,5,22,23,24,25,26]; nephrology sonography guidance for grayscale morphometry and structural kidney assessment [20,21]; and pragmatic standardization measures for patient preparation, bowel-gas reduction, ergonomic setup, and positioning, intended to improve acoustic access and serial comparability [20,21,27,28,29].

The term “standardized” refers to predefined patient preparation, acquisition, calculation, quality-control, and reporting steps intended to reduce avoidable variability. It should not be interpreted as evidence that the complete multi-component procedure has already established universal external validity, feasibility, inter-machine agreement, novice–expert agreement, day-to-day reproducibility, or a validated smallest detectable longitudinal change. Likewise, pragmatic measures such as fasting, bowel-gas reduction, and positioning should not be interpreted as direct evidence that these measures independently improve RRI accuracy or prognostic performance. These implementation properties should be assessed in future prospective studies and through local quality-control programs.

The operational workflow is summarized in Table 1, which provides the minimum acquisition and reporting elements for implementation in outpatient native-kidney CKD. The table is intended as a practical checklist and should be used together with the detailed procedural instructions below.

### 2.2. Patient Selection, Preparation, Positioning, and Same-Visit Clinical Standardization

Adults with native-kidney, non-dialysis-dependent CKD who are clinically stable and able to tolerate the examination are eligible for the standard bilateral protocol. The examination should be deferred or adapted in cases of severe hemodynamic instability, overt acute kidney injury, marked parenchymal distortion, or inability to obtain reproducible intrarenal waveforms, because these factors may compromise interpretation [20,21]. Structural kidney abnormalities, asymmetric renal disease, suspected renovascular disease, limited acoustic windows, inability to maintain the default position, or persistent rhythm irregularity should not automatically preclude acquisition, but they should be documented because they may require protocol adaptation, unilateral or technically incomplete reporting, dedicated renal artery or urinary-tract assessment, or cautious prognostic interpretation [20,21].

An adapted unilateral protocol is appropriate in patients with a solitary native kidney, nephrectomy, nonvisualized kidney, or severe unilateral atrophy, provided that the report states that bilateral mean RRI is not available.

Before scanning, blood pressure and heart rate should be measured after standardized rest using a validated automated device and an appropriately sized cuff. The arm routinely used for clinical follow-up should be used and documented; when a clinically relevant inter-arm difference is known, the arm with the higher pressure should be used for subsequent standardized measurements [30].

An overnight fast of approximately eight hours is preferred when feasible, mainly as an acoustic-window standardization measure extrapolated from abdominal ultrasound and abdominal vascular imaging practice, rather than as direct evidence that fasting improves RRI accuracy [27]. Failure to fast should not invalidate the examination if technically adequate waveforms are obtained. Optional bowel-gas reduction measures, including simethicone or activated charcoal, may be used according to local practice, but their formulation, dose, and timing should be documented when used [28,29]. Water intake should generally be allowed unless clinically contraindicated, because dehydration may alter systemic and renal hemodynamics. Patients should be asked to void before the examination when feasible because collecting-system back-pressure may influence intrarenal Doppler indices [31]. Recent vigorous physical activity, caffeine intake, and nicotine exposure are not protocol exclusion criteria, but they may be recorded when relevant, particularly for serial or research measurements.

Usual medications should not be withheld solely for RRI measurement unless clinically directed. However, the timing of antihypertensive agents, diuretics, renin–angiotensin–aldosterone system (RAAS) inhibitors, sodium–glucose cotransporter 2 (SGLT2) inhibitors, and other vasoactive or nephroprotective treatments should be recorded, and the same timing should be maintained whenever possible for serial examinations.

**Figure 1:**
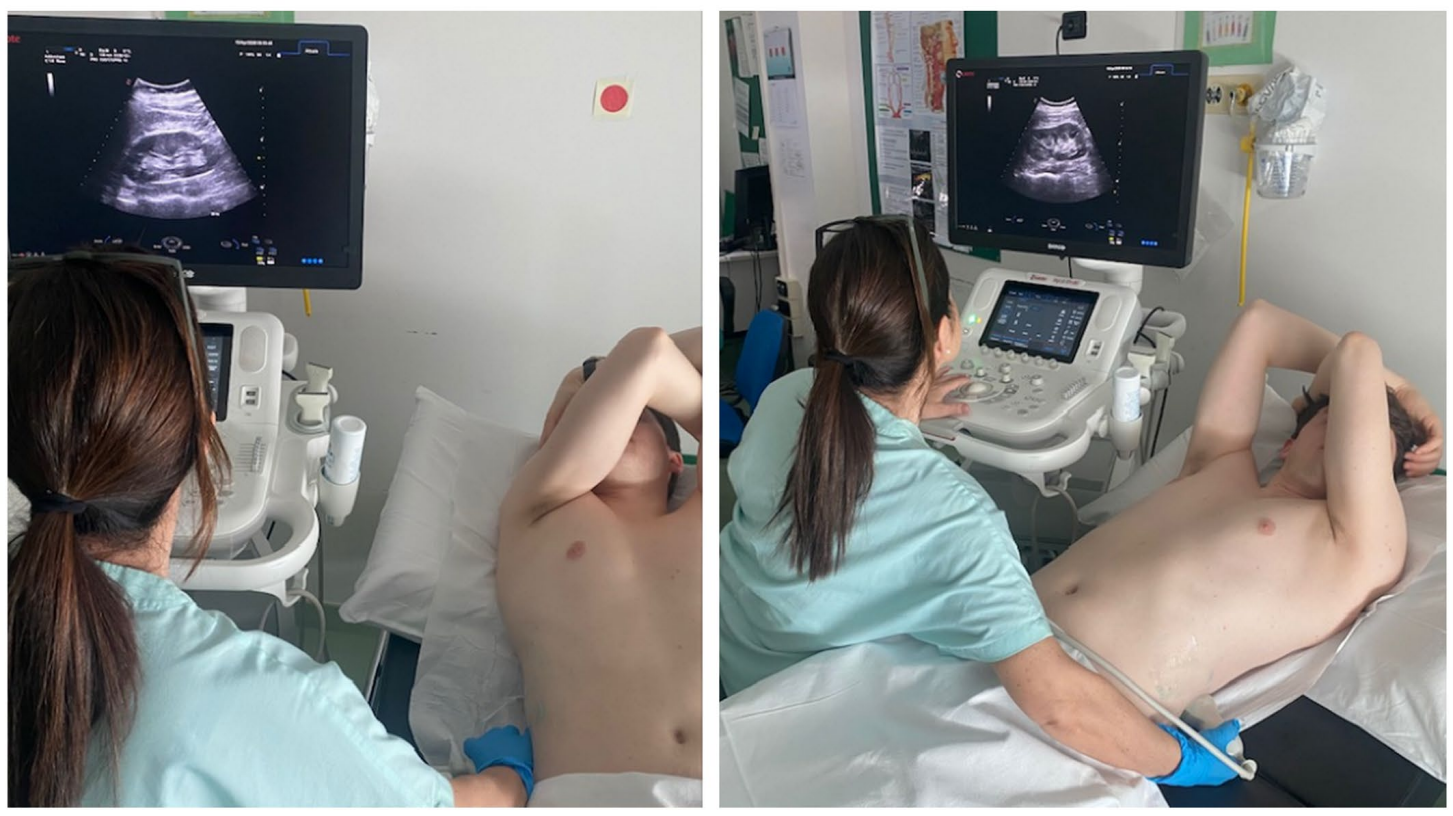
Standardized outpatient setup for renal resistive index measurement. Supine positioning is used as the default starting position, with optional slight rotation, lateral decubitus, posterior intercostal access, prone/ventral positioning, or selective arm elevation when needed to improve the acoustic window. Patient comfort and documentation of the final position are essential for reproducible serial examinations. The illustrated arm position is optional and should be used only when tolerated and helpful for acoustic access.

Supine positioning should be used as the default starting position. A slight contralateral roll, lateral decubitus, posterior intercostal approach, or prone/ventral positioning may be used when needed to improve intrarenal vessel visualization [20,21]. The final position used for each kidney should be documented, especially for serial follow-up. Patient comfort should be prioritized, and arm elevation should be used only when tolerated and when it improves intercostal access (Figure 1).

Before scanning, record the same-visit clinical context. Document the following: (a) KDIGO GFR and albuminuria categories, presumed CKD etiology, and time from first documented CKD diagnosis when available; (b) serum creatinine and estimated GFR, preferably indexed to body surface area and reported as mL/min/1.73 m^2^ using the CKD Epidemiology Collaboration (CKD-EPI) equation adopted by the local laboratory, with the equation version documented when available [12]; (c) urinary albumin- or protein-to-creatinine ratio, or albumin/protein excretion rate; (d) systolic and diastolic blood pressure and heart rate; (e) cardiac rhythm, including atrial fibrillation or frequent ectopic beats; (f) diabetes, hypertension, cardiovascular comorbidity, or anemia; (g) current medications and timing of administration, including vasoactive agents, diuretics, RAAS inhibitors, SGLT2 inhibitors, and other nephroprotective therapies. These variables should be considered during interpretation, particularly for serial examinations.

The time from first documented CKD diagnosis should be interpreted cautiously because it may not reflect the true biological duration of kidney injury; however, it may help contextualize chronicity, treatment exposure, and serial RRI interpretation.

## 3. Materials and Equipment

### 3.1. Ultrasound Equipment Setup and First Grayscale Imaging Acquisition

Use a duplex Doppler ultrasound system equipped with a low-frequency convex-array transducer, typically 2.5–5.0 MHz. Grayscale imaging should precede color and pulsed-wave Doppler acquisition [21,22,24]. Tissue harmonic imaging may be used when it improves corticomedullary definition or renal-margin visualization, particularly in patients with limited acoustic windows [32].

**Figure 2:**
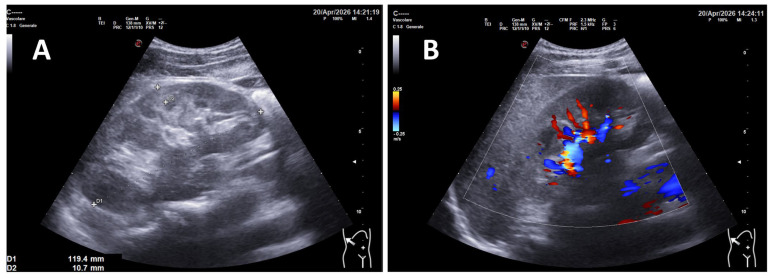
Grayscale and Doppler acquisition sequence. (**A**) Longitudinal grayscale image showing maximum bipolar diameter and cortical thickness measurement, followed by (**B**) color Doppler identification of an interlobar artery adjacent to a medullary pyramid (region of interest, ROI).

Before Doppler acquisition, visualize both kidneys in the longitudinal plane and record maximum bipolar diameter and cortical thickness using standard renal sonography landmarks [20,21] (Figure 2A). Document morphologic findings relevant to interpretation or feasibility, including cortical thinning, poor corticomedullary differentiation, collecting-system dilatation, asymmetry, cystic distortion, or other abnormalities [20,21].

### 3.2. Color Doppler Localization of Interlobar Arteries and Pulsed-Wave Doppler Settings

Color Doppler should be used to identify interlobar arteries adjacent to the medullary pyramids, with the region of interest (ROI) positioned over the target vessel and surrounding parenchyma (Figure 2B) [22,24]. Arcuate arteries may be used when necessary, but the sampling level should be explicitly reported to support reproducibility [3,22,25]. Hilar segmental arteries should not be used for the standard intrarenal RRI protocol.

Doppler settings should be optimized before storing the waveform. The color box (ROI) should be kept as small as practical, color gain should be adjusted to avoid blooming, and the wall filter should remain low enough to preserve end-diastolic flow [5,22,24]. For pulsed-wave Doppler, the sample volume should be placed within a single target vessel and kept small, usually 1–2 mm [5,22,24]. Color and spectral Doppler settings, including pulse repetition frequency (PRF), gain, depth, wall filter, sample volume, and spectral sweep speed, should be adjusted to obtain a sharp spectral envelope with at least three analyzable cardiac cycles [5,22,24].

The Doppler beam should be aligned as parallel to flow as technically feasible, preferably with a displayed angle below 60° when angle correction is used. Although RRI is calculated as the ratio of PSV and EDV from the same trace and is therefore not interpreted as an absolute angle-corrected velocity measurement, adequate beam alignment improves spectral envelope quality and facilitates reliable PSV and EDV identification [4,5,22,25].

In atrial fibrillation or frequent ectopy, additional cycles should be acquired. Ectopic beats, post-ectopic beats, and cycles following long compensatory pauses should be excluded; accepted cycles should have comparable preceding R–R intervals and similar spectral morphology [5,22,24].

## 4. Detailed Procedure

### 4.1. Sampling and Calculation

**Figure 3:**
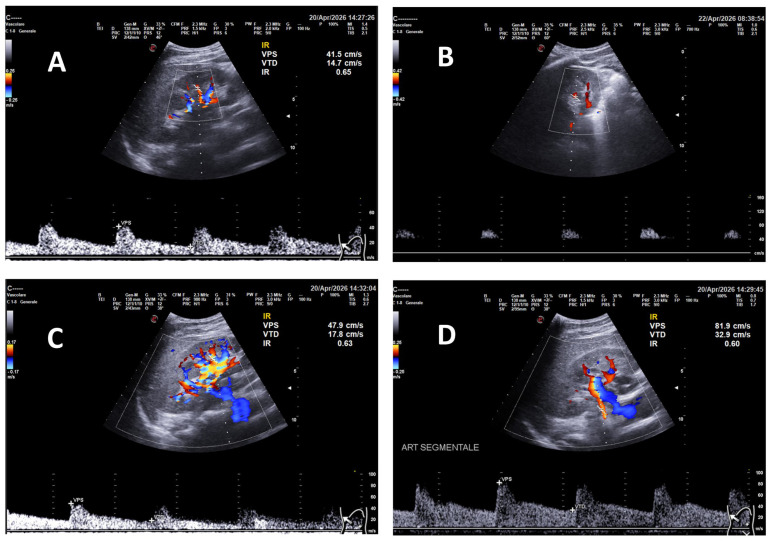
Spectral Doppler waveform analysis and quality control. Device-generated labels are shown as acquired in Italian language. (**A**) Valid interlobar arterial waveform with labeled peak systolic velocity (“VPS” in figure), end-diastolic velocity (“VTD” in figure), and calculated RRI (“IR” in figure). (**B**) Invalid trace due to excessive gain obscuring end-diastolic flow. (**C**) Aliased waveform from inappropriate pulse repetition frequency. (**D**) Off-target trace from inadvertent segmental artery (“art segmentale” in figure) sampling.

Sample the upper, middle, and lower poles of each kidney using the same acquisition logic bilaterally [5,21,26]. At each valid site, measure PSV and EDV from technically acceptable consecutive waveforms and calculate RRI as (PSV − EDV)/PSV [1,2,26]. The site-specific RRI should be calculated as the mean of accepted waveforms, and the kidney-specific mean RRI should be calculated from all valid sites in that kidney (Figure 3A) [26].

A kidney-specific mean should be reported only when at least two of three planned sites are valid. The bilateral mean should be reported only when at least four of six planned sites are valid, including at least two valid sites per kidney. If these criteria are not met, the examination should be reported as technically incomplete, with the number and location of valid sites stated. A unilateral mean may be reported when only one kidney can be adequately sampled. These minimum validity criteria are protocol-defined and intended to avoid reporting poorly sampled examinations as complete studies, while the bilateral multi-site acquisition strategy is consistent with published CKD RRI cohorts and RRI reproducibility-oriented Doppler methodology [18,19,26]. Reported thresholds should be considered pragmatic rather than externally validated.

Before final reporting, review interkidney asymmetry and waveform quality. An absolute side-to-side RRI difference greater than 0.05 or a hilar PSV greater than 2.00 m/s should prompt reassessment of sampling quality and consideration of dedicated renal artery evaluation when clinically appropriate [33,34,35]. Representative grayscale and spectral Doppler images should be archived for each kidney, including additional suboptimal or invalid traces when useful for quality review [21,24].

### 4.2. Quality Control and Troubleshooting

Waveforms should be rejected and reacquired when excessive spectral gain obscures EDV baseline (Figure 3B), when aliasing distorts PSV (Figure 3C), when the sample volume inadvertently includes off-target vessels (Figure 3D), or when respiratory motion prevents stable tracing [5,21,24]. Deep inspiration or an alternative intercostal window may be used during grayscale or color Doppler localization to improve the acoustic window. During spectral acquisition, however, a brief end-expiratory breath-hold is preferred when feasible to reduce kidney motion and stabilize the waveform envelope.

RRI should be interpreted in relation to same-visit hemodynamic conditions because systemic factors may alter PSV, EDV, or both [5,6,7,8,9,23]. Older age, arterial stiffness, widened pulse pressure, and reduced vascular compliance may increase pulsatile transmission and elevate RRI independently of CKD progression [6,7,8,9]. Severe hypotension, tachycardia, dehydration, or acute fluid depletion may reduce end-diastolic forward flow and artifactually increase RRI [6,23]. Atrial fibrillation and frequent ectopy may increase beat-to-beat variability. Right-sided heart failure or venous congestion may alter intrarenal hemodynamics through increased renal venous pressure [36,37], while severe anemia may modify arterial flow profiles through a high-output circulatory state [38]. These conditions should be documented before attributing an elevated or changing RRI to CKD progression.

For serial follow-up, use the same machine, transducer class, preset family, patient position, rest duration, bladder status, and sampling sequence whenever feasible [20,21,22,23,24]. If equipment, preset, or protocol changes are unavoidable, document them and interpret small longitudinal RRI differences cautiously, particularly when they fall within expected intraobserver or interobserver variability [6,10,26,39]. When feasible, a paired same-session comparison using the previous and new equipment or preset may help identify equipment-related offset, although this should be regarded as a pragmatic quality-control measure rather than a validated requirement.

The main technical pitfalls, hemodynamic modifiers, corrective actions, and reporting requirements are summarized in Table 2.

## 5. Expected Results

The expected output of this protocol is a standardized duplex Doppler RRI report in adults with native-kidney non-dialysis-dependent CKD. A technically complete examination should provide kidney-specific mean RRI values for the right and left kidneys and, when validity criteria are fulfilled, an overall bilateral mean RRI. A kidney-specific mean should be reported only when at least two of three planned sampling sites are valid in that kidney, whereas the bilateral mean should be reported only when at least four of six planned sites are valid, including at least two valid sites per kidney. If these criteria are not met, the examination should be classified as technically incomplete, and the number and location of valid sampling sites should be stated. The use of bilateral multi-site averaging is consistent with the acquisition logic of published CKD RRI cohorts and RRI reproducibility-oriented Doppler methodology [18,19,26].

The final report should specify the intrarenal arterial level sampled, the number and location of valid sites, the position used for each kidney, and any deviation from the standard acquisition sequence. It should also include kidney size, cortical thickness, relevant morphologic abnormalities, same-visit blood pressure, heart rate, rhythm, kidney function, albuminuria or proteinuria, and clinically relevant medication or hemodynamic modifiers. When available, estimated GFR should be reported as the KDIGO G category, with the estimated GFR equation specified, and albuminuria/proteinuria should be reported as the KDIGO A category because RRI should be contextualized within the standard CKD risk framework rather than used as an independent staging variable [12].

In a cooperative adult with an adequate acoustic window, the standardized bilateral RRI examination is expected to require approximately 15–20 min, including grayscale morphometry, color Doppler vessel localization, bilateral three-pole spectral acquisition, calculation, and image archiving. Longer acquisition times may be required in patients with obesity, respiratory motion, arrhythmia, distorted renal anatomy, limited acoustic windows, or when additional renal artery assessment is clinically indicated. Operators should have documented training or supervised experience in renal grayscale ultrasonography, intrarenal vascular anatomy, color and pulsed-wave Doppler optimization, PSV and EDV landmark identification, and recognition of invalid waveforms [20,21,22,23,24]. Before clinical implementation for serial follow-up, centers should perform local competency assessment using repeated examinations or repeated stored-image measurements in clinically stable patients. Intraobserver and interobserver variability should be reviewed periodically and after changes in operators, equipment, transducer, or preset. Because universally validated reproducibility thresholds for this complete protocol are not available, local benchmarks should be interpreted in relation to published variability estimates, before interpreting small serial RRI changes as clinically meaningful [6,10,26,39].

To facilitate implementation, a printable operator worksheet and structured reporting template are provided as Supplementary File S1. The worksheet mirrors the minimum acquisition, validity, quality-control, and reporting elements summarized in Table 1 and Table 2 and may be adapted to local clinical or research workflows.

## 6. Discussion

The principal contribution of this protocol is the translation of dispersed renal Doppler methodology into a standardized outpatient CKD workflow. As described in Section 2, this protocol should be regarded as a proposed standardized technical workflow rather than as an externally validated multi-center procedure. By defining patient preparation, grayscale morphometry, intrarenal vessel targeting, bilateral three-pole acquisition, waveform rejection criteria, averaging rules, minimum validity thresholds, and structured reporting, the protocol aims to reduce preventable acquisition variability and improve comparability across operators and serial examinations [20,21,22,23,24,26]. This is particularly important because RRI is clinically useful only when interpreted as a contextual hemodynamic and prognostic marker rather than as an isolated diagnostic threshold [5,13,15,16,17,18,19].

Standardization is especially relevant for serial follow-up. RRI is less dependent on Doppler angle than absolute velocity measurements because PSV and EDV are obtained from the same spectral trace; however, consistent sampling territory and waveform quality remain essential [4,5,22,25]. Interlobar or arcuate arteries are preferred because they are intraparenchymal sampling sites and are commonly used to capture Doppler signals related to nephroparenchymal and microvascular damage [5,9,13,15]. Segmental or hilar sampling should not be mixed with the standard protocol unless explicitly documented, because sampling level affects comparability and published studies have used different intrarenal arterial targets [18,22,26,40,41]. Reproducibility estimates from prior studies suggest that small serial differences should be interpreted cautiously, particularly when acquisition conditions differ [6,10,26,39].

RRI should be interpreted within the KDIGO GFR and albuminuria framework rather than as an independent CKD staging tool [12]. Higher RRI has been associated with lower GFR, histologic chronicity, proteinuria-related risk, CKD progression, dialysis initiation, and mortality [13,14,15,16,17,18,19,40], while age, pulse pressure, arterial stiffness, diabetes, diabetic kidney disease, and cardiovascular disease may substantially modify its interpretation [5,7,8,9,42,43]. Kidney morphology, including cortical thickness, should also be considered when interpreting RRI in CKD [20,21]. However, RRI lacks etiologic specificity, has only modest discrimination as a single prognostic marker, and is not included in KDIGO recommendations for CKD staging or prognosis assessment [5,12,18,21]. Therefore, it should complement, not replace, GFR, albuminuria, kidney morphology, and clinical judgment [5,12,21].

Future outpatient kidney ultrasound protocols may integrate RRI with complementary approaches that better characterize renal congestion, perfusion, and microvascular structure. Intrarenal venous Doppler and venous congestion indices may help distinguish arterial impedance from venous-pressure-related renal dysfunction, particularly in cardiorenal phenotypes [36,37]. Ultrasound kidney morphometry, including kidney size and potentially volume assessment, may also refine structural phenotyping in CKD [20,21]. In addition, contrast-enhanced ultrasound may allow for quantitative assessment of renal cortical microperfusion, while super-resolution ultrasound may provide more detailed visualization of renal microvascular architecture [44,45,46]. These techniques should currently be considered complementary or investigational in relation to standardized RRI measurement, pending broader validation in outpatient CKD cohorts.

## Figures and Tables

**Table 1:** Operational workflow and reporting checklist for outpatient RRI measurement in native-kidney CKD.

Protocol Domain	Minimum Standard	What to Report
Eligibility and preparation	Adult with native-kidney, non-dialysis-dependent CKD; clinically stable; able to tolerate the examination. Prefer 5 min quiet rest, voiding when feasible, and approximately 8 h fasting when acoustic-window standardization or broader vascular assessment is desired.	State whether the standard bilateral protocol was completed. Document fasting/voiding status and any deviation from standard preparation.
Same-visit clinical context	Record estimated GFR category, specifying the estimated GFR equation indexed to BSA, such as the CKD-EPI equation adopted and its version when available, albuminuria or proteinuria category, BP, HR, rhythm, hydration status, CKD etiology when known, relevant comorbidities, hemoglobin if available, and vasoactive/nephroprotective medications including the timing of administration.	Report the clinical and hemodynamic context alongside RRI and hydration status; include approximate time from CKD diagnosis and previous estimated GFR trajectory when available. Report estimated GFR in mL/min/1.73 m^2^ and recent caffeine/nicotine/exercise exposure when relevant for serial or research measurements.
Patient positioning	Start supine for standardization. Use slight oblique rotation, lateral decubitus, posterior intercostal, or prone/ventral positioning when needed to improve intrarenal visualization. Arm elevation should be optional and comfort-dependent.	Document the final position and approach for each kidney, especially for serial follow-up.
Grayscale assessment	Before Doppler, measure kidney length and cortical thickness bilaterally and assess morphology.	Report kidney size, cortical thickness, cortical thinning, poor corticomedullary differentiation, collecting-system dilatation, asymmetry, cystic distortion, or other relevant abnormalities.
Target vessel and Doppler setup	Prefer interlobar arteries adjacent to medullary pyramids. Use arcuate arteries only if explicitly documented. Optimize color ROI, gain, PRF, wall filter, sample volume, and spectral sweep before storing traces.	Specify vessel level, non-standard sampling, probe approach, and any technical limitation. Do not mix hilar/segmental measurements with the intrarenal protocol.
Per-site acquisition	Sample upper, middle, and lower poles in each kidney. Acquire at least 3 comparable cycles per site; use more cycles in atrial fibrillation or ectopy and exclude ectopic/post-ectopic cycles.	Report number and location of valid sites and any reduced cycle count due to rhythm or technical limitations.
Calculation and validity	Calculate per-waveform RRI = (PSV − EDV)/PSV; site mean = mean of accepted cycles. Kidney mean requires at least 2 valid sites in that kidney; bilateral mean requires at least 4 valid sites total, including at least 2 per kidney.	Provide right, left, and bilateral mean RRI when valid. If criteria are not met, classify as technically incomplete and explain missing data.
Asymmetry and renal artery trigger	Before sign-out, review interkidney RRI difference >0.05 or hilar PSV > 2.0 m/s if assessed.	Annotate persistent asymmetry and consider dedicated renal artery assessment when clinically indicated.
Image archiving and serial follow-up	Save representative grayscale morphometry and valid spectral traces for each kidney. Use the same machine, preset family, transducer class, position, and sequence for follow-up whenever possible.	Document saved representative images and any machine, preset, protocol, positioning, or completeness change between serial studies.
Integrated interpretation	Interpret RRI as an adjunctive hemodynamic/prognostic marker, not a stand-alone diagnostic test or CKD staging tool.	Integrate RRI with estimated GFR, albuminuria/proteinuria, morphology, age, pulse pressure, rhythm, volume status, anemia, cardiovascular disease, and medications.

RRI, renal resistive index; CKD, chronic kidney disease; BSA, body surface area; CKD-EPI, chronic kidney disease epidemiology collaboration; PSV, peak systolic velocity; EDV, end-diastolic velocity; GFR, glomerular filtration rate/estimated glomerular filtration rate; BP, blood pressure; HR, heart rate; PRF, pulse repetition frequency; ROI, region of interest.

**Table 2:** Quality-control and interpretation checklist for RRI measurement.

Issue	Likely Effect on Waveform or RRI	Action/Reporting Requirement
EDV not reliable	EDV may be obscured or suppressed, producing spuriously high or indeterminate RRI.	Lower gain and wall filter, optimize baseline, and reacquire. If EDV remains undetectable after optimization, mark the site invalid.
PSV not reliable	PSV may be truncated or distorted; calculated RRI becomes unreliable.	Adjust PRF/depth/gain and reacquire until the systolic peak is clearly displayed.
Mixed or off-target sampling	Composite or non-comparable waveform; interlobar/arcuate interpretation no longer applies.	Use a small gate within a single interlobar artery adjacent to a medullary pyramid. If arcuate sampling is used, state this explicitly.
Motion, poor acoustic window, large color box, or distended bladder	Unstable envelope, poor vessel localization, or possible artifactually increased RRI.	Use brief end-expiratory breath-hold, alternative window/position, reduced color ROI, and voiding when feasible. Document limitations.
Interkidney RRI difference >0.05 or hilar PSV > 2.0 m/s if assessed	May indicate technical error, asymmetric parenchymal disease, or unilateral renal artery stenosis.	Review technique and waveform quality bilaterally. If discrepancy persists, report it and consider dedicated renal artery assessment when clinically appropriate.
Atrial fibrillation or frequent ectopy	Beat-to-beat PSV/EDV variability may destabilize site means.	Acquire at least 5 cycles; exclude ectopic/post-ectopic cycles and cycles after long pauses; average comparable cycles and report rhythm-related limitations.
Low-flow or acute hemodynamic states	Reduced end-diastolic forward flow may artifactually increase RRI independent of CKD progression.	Record same-day BP, HR, and volume context. Avoid over-attributing elevated RRI to chronic parenchymal disease.
Right heart failure or venous congestion	Increased renal venous pressure may alter intrarenal hemodynamics and reduce effective perfusion gradient.	Annotate congestion/right-heart status; interpret RRI with cardiorenal context and consider complementary venous Doppler indices when appropriate.
Systemic factors modifiers	RRI may be elevated or waveform profile altered independently of CKD stage or etiology	Integrate RRI with age, kidney morphology, estimated GFR, albuminuria/proteinuria, pulse pressure, cardiovascular burden, and hemoglobin when available.
Serial-study non-comparability	Small longitudinal changes may reflect acquisition or equipment differences rather than biological progression.	Document all changes. Prefer the same setup and, if feasible, paired bridging measurements before interpreting small interval shifts.

RRI, renal resistive index; CKD, chronic kidney disease; PSV, peak systolic velocity; EDV, end-diastolic velocity; GFR, glomerular filtration rate/estimated glomerular filtration rate; BP, blood pressure; HR, heart rate; PRF, pulse repetition frequency; ROI, region of interest.

## Data Availability

No new clinical cohort dataset was generated or analyzed in this Protocol article. The illustrative photographs and ultrasound images were obtained from consenting adult volunteers and/or de-identified representative examinations solely to demonstrate patient positioning, acquisition, and quality-control principles. These images are included in the manuscript; no additional analyzable patient-level dataset is associated with this article.

## Supplementary Materials

The following supporting information can be downloaded at https://www.mdpi.com/article/10.3390/diagnostics16152438/s1, Supplementary File S1: Operator worksheet and structured reporting template.
