# Supplementary material for: Duplex Doppler Measurement of Renal Resistive Index in Non-Dialysis Chronic Kidney Disease Outpatients: A Proposed Standardized Protocol
Source: Diagnostics (Basel). 2026 Aug 1;16(15):2438. doi: 10.3390/diagnostics16152438 (PMC13464445; doi:10.3390/diagnostics16152438)
Supplement: Supplementary file 1 [file diagnostics-16-02438-s001.zip › diagnostics-4435305-supplementary.pdf]

Supplementary File S1. Operator worksheet and structured reporting template

Duplex Doppler measurement of renal resistive index (RRI) in native-kidney non-dialysis CKD

Purpose: Optional compact clinic workflow template for recording minimum acquisition, validity, quality-control, and reporting elements. Adapt to local clinical or research requirements.

A. Examination, preparation, and clinical context

Item	Entry / check
**Date / operator**	Date: ____________________ Operator: ____________________
**System / transducer / preset**	System: ____; Transducer: ____________________;<br>Preset: ____;
**Study type**	[ ] First examination; [ ] Serial follow-up;<br>Prior setup changed: [ ] No, [ ] Yes, specify: ____;
**Eligibility and preparation**	[ ] Native-kidney non-dialysis CKD; Clinically stable: [ ] Yes, [ ] No, specify:____________________;<br>[ ] Voided; [ ] Fasted (~8 h); Prior [ ] physical activity, [ ] caffeine intake, or [ ] nicotine exposure;<br>Hydration status: [ ] poor, [ ] good, [ ] overload, specify:____;
**Positioning**	Default supine. Final position/approach should be recorded for each kidney below.<br>Specify: ____;
**CKD context**	Etiology: ____;<br>Time from first documented CKD diagnosis: ____;<br>eGFR trajectory: ____;
**Kidney function and albuminuria**	Creatinine: ________; eGFR:_________, BSA indexed to 1.73 mL/min/1.73 m^2: [ ] Yes, [ ] No;<br>Equation/version:______; KDIGO G: ___; Albuminuria/proteinuria: ____(units: ____); KDIGO A:__;
**Same-visit hemodynamics**	BP: ______/______ mmHg; HR: ______ bpm;<br>Rhythm: [ ] Sinus, [ ] AF, [ ] Ectopy, [ ] Other: ____;
**Comorbidities and modifiers**	[ ] Diabetes; [ ] Hypertension; [ ] Cardiovascular disease; [ ] Anemia;<br>[ ] Venous congestion/right heart failure; [ ] Volume depletion;
**Relevant medication**	[ ] RAAS inhibitor; [ ] SGLT2 inhibitor; [ ] Diuretic; [ ] Vasoactive agent;<br>[ ] Recent medication change: ____;<br>Timing of administration: ____;

B. Kidney morphology and acquisition approach

Kidney	Length (mm)	Cortical thickness (mm)	Relevant morphology	Position / approach	Image saved
**Right**	_________________	__________________	______________________<br>______________________	_______________________<br>_______________________	[ ] Yes
**Left**	_________________	__________________	______________________<br>______________________	_______________________<br>_______________________	[ ] Yes

C. RRI acquisition and calculation

Formula: RRI = (PSV - EDV) / PSV. Use comparable technically valid cycles. In AF/ectopy, exclude ectopic/post-ectopic cycles and cycles after long pauses.

Kidney	Pole	Vessel level	RRI cycles or mean values	Site valid?	Site mean	Comments
**Right**	**Upper**	[ ] Interlobar;<br>[ ] Arcuate;	1: ____, 2: ____, 3: ____,<br>extra: ________________;	[ ] Yes, [ ] No	_____________	_______________
**Right**	**Middle**	[ ] Interlobar;<br>[ ] Arcuate;	1: ____, 2: ____, 3: ____,<br>extra: ________________;	[ ] Yes, [ ] No	_____________	_______________
**Right**	**Lower**	[ ] Interlobar;<br>[ ] Arcuate;	1: ____, 2: ____, 3: ____,<br>extra: ________________;	[ ] Yes, [ ] No	_____________	_______________
**Left**	**Upper**	[ ] Interlobar;<br>[ ] Arcuate;	1: ____, 2: ____, 3: ____,<br>extra: ________________;	[ ] Yes, [ ] No	_____________	_______________
**Left**	**Middle**	[ ] Interlobar;<br>[ ] Arcuate;	1: ____, 2: ____, 3: ____,<br>extra: ________________;	[ ] Yes, [ ] No	_____________	_______________
**Left**	**Lower**	[ ] Interlobar;<br>[ ] Arcuate;	1: ____, 2: ____, 3: ____,<br>extra: ________________;	[ ] Yes, [ ] No	_____________	_______________

Validity and summary

Right kidney	Left kidney	Bilateral dataset	Final classification
Valid sites: ____/3;<br>Mean RRI: ____;	Valid sites: ____/3;<br>Mean RRI: ____;	Valid sites: ____/6;<br>Bilateral mean RRI: ____;	[ ] Complete;<br>[ ] Technically incomplete;<br>Reason: ____;

Minimum validity rule: Report kidney-specific mean only if >=2/3 sites are valid in that kidney. Report bilateral mean only if >=4/6 sites are valid, including >=2 valid sites per kidney.

D. Quality control and structured interpretation

Quality-control item	Check / note
**PSV and EDV clearly identifiable**	[ ] Yes , [ ] No; reacquired/invalid site: ______________________________;
**No aliasing, excessive gain, high wall filter, mixed vessel, or unstable respiratory trace**	[ ] Yes, [ ] No; limitation: ____;
**AF/ectopy cycle selection applied when needed**	[ ] Not applicable, [ ] Yes, [ ] Limited by rhythm;
**Interkidney RRI difference >0.05 or hilar PSV >2.0 m/s if assessed**	[ ] No, [ ] Yes;<br>renal artery assessment considered: [ ] No, [ ] Yes;
**Major hemodynamic modifiers present**	[ ] No, [ ] Yes; specify: ____:
**Serial comparability**	[ ] Same setup; [ ] Setup changed;<br>Specify machine/preset/position/completeness change:<br>____;

Final structured report

RRI examination: [ ] Complete [ ] Technically incomplete
**Right mean RRI:** ______ **Left mean RRI:** ______ **Bilateral mean RRI:** ______
Main morphologic context: ____
Hemodynamic / clinical caveats: ____
Technical limitations: ____
**Interpretation:** RRI is an adjunctive hemodynamic/prognostic marker and not a stand-alone diagnostic test or CKD staging tool.

Abbreviations: RRI, renal resistive index; CKD, chronic kidney disease; BSA, body surface area; PSV, peak systolic velocity; EDV, end-diastolic velocity; eGFR, estimated glomerular filtration rate; BP, blood pressure; HR, heart rate; AF, atrial fibrillation; PRF, pulse repetition frequency; ROI, region of interest; RAAS, renin-angiotensin-aldosterone system; SGLT2, sodium-glucose cotransporter-2.
